# Saccadic gain adaptation is predicted by the statistics of natural fluctuations in oculomotor function

**DOI:** 10.3389/fncom.2012.00096

**Published:** 2012-12-06

**Authors:** Mark V. Albert, Nicolas Catz, Peter Thier, Konrad Kording

**Affiliations:** ^1^Sensory Motor Performance Program, Rehabilitation Institute of ChicagoChicago, IL, USA; ^2^Department of Physical Medicine and Rehabilitation, Northwestern UniversityChicago, IL, USA; ^3^Laboratory of Adaptive and Integrative Neuroscience, Fédération de Recherche 3C, CNRS and University of Aix-MarseilleMarseille, France; ^4^Department of Cognitive Neurology, Hertie Institute for Clinical Brain Research, Eberhard Karls Universität TübingenTübingen, Germany

**Keywords:** oculomotor system, cerebellar vermis, saccade adaptation, natural statistics, multiple-timescale adaptation

## Abstract

Due to multiple factors such as fatigue, muscle strengthening, and neural plasticity, the responsiveness of the motor apparatus to neural commands changes over time. To enable precise movements the nervous system must adapt to compensate for these changes. Recent models of motor adaptation derive from assumptions about the way the motor apparatus changes. Characterizing these changes is difficult because motor adaptation happens at the same time, masking most of the effects of ongoing changes. Here, we analyze eye movements of monkeys with lesions to the posterior cerebellar vermis that impair adaptation. Their fluctuations better reveal the underlying changes of the motor system over time. When these measured, unadapted changes are used to derive optimal motor adaptation rules the prediction precision significantly improves. Among three models that similarly fit single-day adaptation results, the model that also matches the temporal correlations of the non-adapting saccades most accurately predicts multiple day adaptation. Saccadic gain adaptation is well matched to the natural statistics of fluctuations of the oculomotor plant.

## Introduction

Our movement system changes due to a large number of factors including fatigue, disease, attention, nutrition, exercise, growth, and depletion of neurotransmitters. Recent models of motor adaptation have been derived based on the idea that movement adaptation serves to undo such changes to maintain stable responses to movement commands (Korenberg and Ghahramani, 2002; Kording et al., 2007; Burge et al., 2008; van Beers, 2009; Wei and Koerding, 2009; Shadmehr et al., 2010). These models assume how the movement system might fluctuate over time and derive the optimal solution to the adaptation problem. These results often demonstrate good fits to experimentally measured adaptation behavior.

To test the hypothesis that motor adaptation is well matched to the way the motor system actually changes it is necessary to measure those changes. Normally, adaptation happens concurrently with ongoing fluctuations in the responsiveness of the neuromotor plant. For example, when muscles fatigue, the signal sent to the motor plant increases to compensate for that fatigue. When muscles become stronger, or energy is more plentiful, movement commands are also modulated to perform the same reach movement. Under normal circumstances, this ongoing adaptation largely masks the effects of underlying changes to the motor system (Shelhamer and Joiner, 2003). For this reason, it has never been quantitatively tested if adaptation is matched to ongoing fluctuations in the responsiveness of the motor plant.

The oculomotor system is a particularly popular model system to study motor adaptation. Analysis is simplified because saccades are primarily ballistic; they happen too quickly for proprioceptive feedback to strongly affect the saccade (Keller and Robinson, 1971; Guthrie et al., 1983). Saccade adaptation has been well-studied through both behavioral and neurophysiological experiments (McLaughlin, 1967; Miller et al., 1981; Deubel et al., 1986; Semmlow et al., 1989; Frens and Van Opstal, 1994; Straube and Deubel, 1995; Fuchs et al., 1996; Thier et al., 2002; Hopp and Fuchs, 2004; Robinson et al., 2006). In a typical saccade adaptation experiment, the saccade target is systematically moved mid-saccade, and after learning the subject begins to saccade to the predicted location of the target rather than the initial location. It has been shown that saccadic gain adaptation can largely be abolished by lesions in the oculomotor region of the cerebellar vermis (Optican and Robinson, 1980). For this reason, saccade adaptation provides a model system for studying motor adaptation and its link to normal disturbances in the motor plant.

Here we use monkeys with lesions in the cerebellar vermis, with severe deficits in adaptation, to measure the ongoing changes in the oculomotor system. Based on this data we construct a model with simulated behavior that matches measured changes of the oculomotor plant. Because of the fit to this data, the models have fewer free parameters. Interestingly, this reduction of the number of free parameters leads to a superior prediction precision for multiday saccade adaptation. Because of this predictive ability, it appears that the movement adaptation system is well matched to the statistics of naturally occurring fluctuations in the responsiveness of the oculomotor system.

## Methods

**Figure 1:**
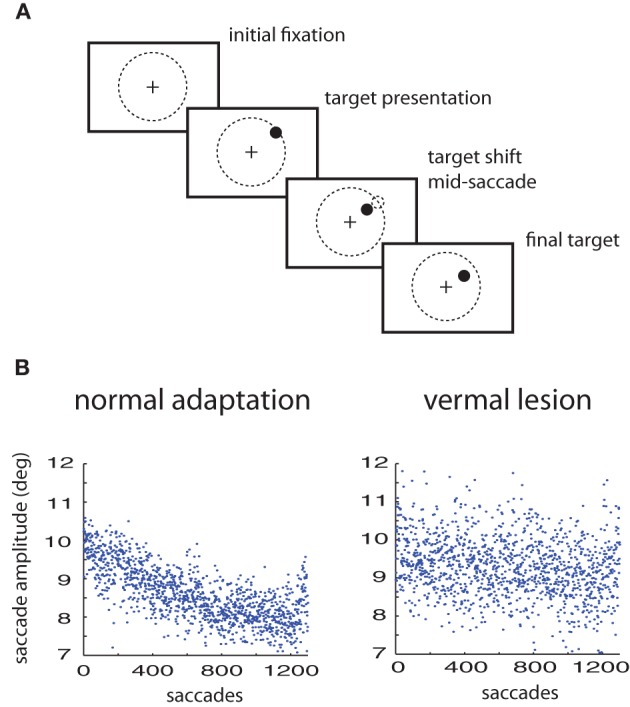
**Saccade adaptation paradigm. (A)** Experimental design: a saccade target is presented. Mid-saccade the target is shifted. Prior to training, this results in expected errors. After training, the monkey saccades directly to the shifted target location. **(B)** Saccade adaptation to a 3° shift of the target in a normal animal compared to the same animal with a lesion in the cerebellar vermis.

All procedures complied with the National Institutes of Health Guide for Care and Use of Laboratory Animals and were approved by the local animal care committee (RP Tubingen, FG Tierschutz). The experimental design is a standard saccade adaptation paradigm using two macaques; specific details are available in Thier et al. (2002). After maintaining fixation a target is presented at 10° in the periphery. The monkey is instructed to saccade to the target. The target is shifted during the saccade to either a 7° or 13° eccentricity (see Figure 1A). Recordings were performed both before and after lesions of the posterior cerebellar vermis. The results were modeling using variants of the general multiple timescale adaptation model of Kording, Tenenbaum, and Shadmehr (Kording et al., 2007)—referred to here as the KTS model.

### Adaption model

The driving assumption of our adaptation model is that we must estimate continuously changing disturbances to the oculomotor plant. We model the manner in which these disturbances change as a sum of random walks at different timescales. Specifically, each disturbance to the motor plant is modeled by Equation 1. Each disturbance is associated with a specific exponential decay time constant τ. A larger τ implies the disturbance changes slowly, whereas a smaller τ increases the relative effect of noise and implies a faster timescale, as evident in Equation 1.

(1) $${\text{disturbance}}_{\tau }\left(t+1\right)=\left(1-1/\tau \right)\times {\text{disturbance}}_{\tau }\left(t\right)+\text{process noise }(0,\sigma_{\tau })$$

To approximate a continuous distribution of timescales, 30 separate time constants were chosen uniformly within the range appropriate for saccade experiments (2 and 3.3 × 10^5^ saccades). The process noise is drawn from a normal distribution with mean 0 and width σ_τ_. Here we assume this noise scales inversely with the time constant, τ, and is specified explicitly by Equation 2. Note in the KTS model, *a* = 1.

(2) $$\sigma_{\tau }=c\tau^{-a}$$

For these individual, timescale-dependent disturbances, we can determine the overall gain of the system. The gain is simply the sum of the disturbances over these different timescales.

(3) $$\text{gain}\left(t\right)=1+\sum_{\tau }{\text{disturbance}}_{\tau }\left(t\right)$$

Unfortunately, we never observe the intended gain of the system directly—there is always observation noise. In the case of the oculomotor system, the noise in the observation can come from both the lack of precision in measurement, but more importantly from an inability of the motor plant to transmit and produce the same movement given the same internal command.

(4) $$\text{observation}\left(t\right)=\text{gain}\left(t\right)+\text{observation noise }(0,\sigma_{w})$$

Because the model has been clearly defined, an optimal adaptation strategy can be derived. We note that this sytem is equivalent to the generative model of a Kalman filter with the following properties:

- A diagonal transition matrix, M = diagonal(1 − τ^−1^)
- An observation matrix, H = Identity (30 × 30 for each of the disturbances)
- A diagonal process noise matrix, Q = diagonal(c τ^−*a*^)
- A state vector, x of the 30 disturbances

We used the Kalman filter toolbox (written by K. Murphy, University of British Columbia, Vancouver, Canada) to solve these equations and determine the optimum sum and distribution of gains for each simulated experiment. The parameters used to best fit the saccade behavior are described below.

### The three specific models

In this paper, we contrast three different models based on the original multiple timescale adaptation model (Kording et al., 2007).

#### The original KTS model

In their original paper, the free parameters were not rigorously selected, as the same approximate learning curve can be derived from a change in either of the noise parameters—process or observation noise. The free parameters used in that work (*c* = 0.001^2^, *a* = 1, σ_*w*_ = 0.05) were chosen as they yielded qualitatively accurate fits to the data, but were not directly estimated. The other two model variants fit these free parameters to the experimental data.

#### The equal variance model

We consider the KTS model, with *a* = 1 in the distribution of process noise, but allow for the other parameters to be fit given the procedure detailed below. This is the equal variance model because the form of the process noise matrix dictates an equal contribution to the variance for each timescale.

#### The weighted variance model

To be able to accurately fit the autocorrelation function as detailed below, we required greater flexibility in the distribution of process noise across the different timescales. This model essentially allows “*a*” to be a free parameter. By allowing this, we were able to accurately fit the autocorrelation function.

### Model fitting procedure

The parameters for each model were fit according to the following procedure and are given in Table 1. For the equal and weighted variance models, “*c*” and “*a*,” representing the distribution of system noise, were found by fitting data from the lesioned animal behavior. σ_*w*_, the observation noise, was chosen by fitting to the one-session data in intact primates.

**Table 1:** **Model parameters**.

**Model**	**σ_*w*_**	***c***	***a***
Original KTS	0.05	1.475 × 10^−6^	1
Equal variance	0.65	3.5 × 10^−4^	1
Weighted variance	0.8	9.1 × 10^−5^	0.7

**Figure 2:**
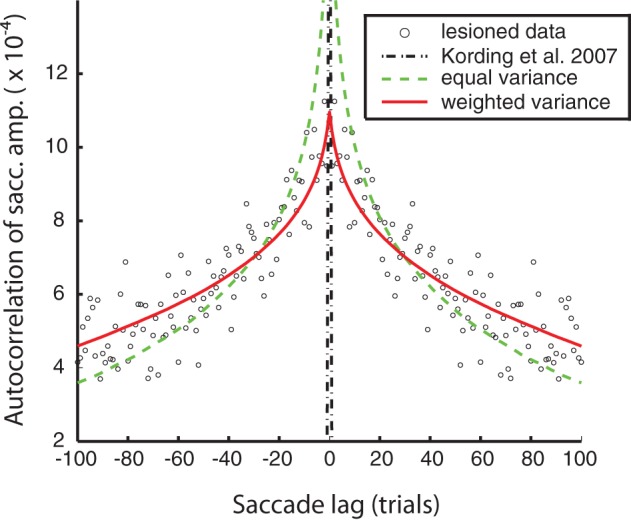
**Model fits to the lesioned saccade autocorrelation function (black circles).** Fits are based on autocorrelations from model saccades in an unperturbed simulated experiment. Black, mixed: the original free parameter choices from the Kording et al. (2007) model—negligible autocorrelation due to high uncorrelated observation noise. Green, dashed: equal process noise variance model (see “Methods” for details). Red, solid: the weighted process noise variance model.

The goal of the autocorrelation fits for the non-adapting animals was to capture the timescales of natural fluctuations in motor responsiveness. The data used were the saccade errors in the lesioned monkey saccade adaptation sessions. The autocorrelation of the saccade errors was performed using the unbiased cross-correlation function provided by Matlab. The overall autocorrelation function, to which the fits were measured, was produced by averaging the autocorrelation functions from each lesioned saccade adaptation session for both monkeys. This procedure produced the saccade data used for fitting (shown in Figure 2). The lag in the autocorrelation was cut off at 100 saccades, both for presentation purposes and fitting.

We were able to obtain noise-free autocorrelation functions from the model variants by setting observation noise to zero and observing the behavior of the system over time. The original KTS free parameter choices substantially failed to match the lesioned animal autocorrelation data; this is because the largest source of error was from observation noise, which is uncorrelated between trials and so leads to a spike in the autocorrelation function at zero. For the two model fits, we adapted at least one free parameter in this case, “*c*,” to fit the autocorrelation function of the lesion behavior. We observe that using the original KTS formulation could not adequately model the autocorrelation function. The autocorrelation fit was improved by the introduction of the additional parameter “*a*” in the weighted variance model.

To set the final parameter, the observation noise, we fit the models to one-session saccade adaptation data recorded prior to the lesion. The original KTS parameter model is shown for comparison; though its observation noise wasn't fit to this specific data, it provided a fairly reasonable fit. For the other models the observation noise was adjusted to give the optimal mean squared error. By fitting to both the lesioned and unlesioned behavior, we were capable of capturing the trend of a single-session reasonably well.

The experimental multiple-session data was from Robinson et al. (2006). At night the animals were kept in the dark. This was done so their saccades could not adapt back to normal between sessions. The period between sessions was simulated as 1500 unobserved saccades; this parameter was robust to deviations, and so this value was set arbitrarily. As we had no access to the originally published data used for Figure 4 (Robinson et al., 2006), the data was extracted from the original figure. Artifacts that did not represent data, such as tic marks, regression lines, and grid lines, were removed and the image was smoothed using a Gaussian filter with a standard deviation equal to approximately the circular size of the data points. For every 100 saccades (60 total locations along the horizontal axis) the distribution of saccades was approximated based on the presence of non-white pixels at the appropriate scaling. The vertical mean and variance for those saccades were calculated by weighing the contribution for each pixel by the luminance difference from white. Figure 4 was generated using Gaussians from the previously calculated means and variances to provide the background for the model overlays—higher values were darker.

The three models were compared using the Bayesian Information Criteria (BIC) estimate for model selection (Schwarz, 1978) to account for the different number of free parameters in the models. Using the means and variances from the data used to generate Figure 4, the log likelihood was calculated for each model. Bootstrap resampling was used on the pixels to calculate confidence intervals for the log likelihoods. The number of free parameters in the BIC calculation are *k* = 0 for the Kording et al. model (it wasn't fit to the data presented here), *k* = 2 for the equal variance model, and *k* = 3 for the weighted variance model.

### Saccade recordings and vermal lesions

A brief summary of the lesions and procedure are given below. A more complete description of the vermal lesions and adaptation procedure has been previously reported (Ignashchenkova et al., 2009).

Two male rhesus monkeys (*Macaca mulatta*; referred to as monkeys B and R) were implanted with a scleral search coil and a head post for painless head restraint. All procedures complied with the National Institutes of Health Guide for Care and Use of Laboratory Animals and were approved by the local animal care committee (RP Tübingen, FG Tierschutz). The monkeys were positioned 22 cm away from the computer monitor. Eye movements were monitored during all the tasks using the search coil technique (spatial resolution 0.1° of visual angle, temporal resolution 1 kHz). Deviations of eye position from the fixation point exceeding a certain sized bounding box were excluded from the analysis (preoperatively 2–2.5°, postoperatively 4–8°).

Animals were trained to make visually guided saccades to targets at an eccentricity of 7 or 13° in eight different directions. A fixation point appeared in the center of the screen for 500 ms, followed by the disappearance of the fixation point and the simultaneous appearance of a saccade target, which was shown for 700 ms. Monkeys were trained to make precise saccades to the target for a fluid reward that they received if they moved their eyes to the target within 400 ms after its appearance.

The lesions were centered on somewhat different parts of the posterior vermis, but both initially showed the expected saccade deficits immediately after the lesion. At the completion of the experiments (3 mo for monkey R, 6.5 mo for monkey B) the monkeys were deeply anesthetized and perfused. The cerebellum was sectioned parasagittally and Nissl stained. Monkey B and R exhibited hypometria of saccades soon after lesioning, and had complete or partial lesions of lobuli V–VIII. Specifically, Monkey B had partial ablations of lobulus V and the fastigial nucleus and complete ablations of lobuli VI–VIII, and monkey R had partial ablations of lobuli V–VIII. These lesions and the effects are further documented elsewhere (Ignashchenkova et al., 2009).

## Results

Healthy monkeys respond to artificially imposed errors in their eye movements by increasing or decreasing the gain over durations of hundreds of their saccades so that their errors get reduced (Figure 1A). However, with lesions of the posterior cerebellar vermis the ability of these monkeys to adapt their saccade gains based on errors is severely impaired, at least in the timescales of the experiment (Figure 1B). The responses of the oculomotor system continue to fluctuate but adaptation is much weaker or non-existent (Thier et al., 2002). Here we will use the measured fluctuations of lesioned monkeys to improve predictions on the adaptation curves of healthy monkeys.

We can characterize these fluctuations in motor response using the autocorrelation function of eye-movement errors. A peak in the center indicates a correlation between errors in nearby trials. In humans, it is known that under normal conditions this autocorrelation function is flat, indicating that the adaptation system removes temporal correlations that might exist in the errors (Shelhamer and Joiner, 2003). However, for the lesioned monkeys, while they show greatly diminished adaptation they still show a non-flat autocorrelation function, indicating that subsequent eye-movements are not independent from one another (Figure 2). In viewing the autocorrelation function, we see that there is higher correlation in errors between successive saccades, and that correlation declines characteristically as the distance between saccade trials increases. Importantly, errors made due to ongoing changes in the oculomotor system persist, making subsequent eye movements correlated. This autocorrelation function thus characterizes the temporal scales over which the oculomotor system naturally fluctuates.

We first wanted to test if the previous model of Kording et al. (2007) accurately described the measured non-adapting oculomotor fluctuations. We plot the autocorrelation function predicted by that model along with the actual autocorrelation function (Figure 2). We find that for the parameter values used in that paper predicted an autocorrelation function for these changes that is very different from what we observed experimentally. This finding highlights the importance of quantifying the way the system changes over time instead of making assumptions.

What assumptions about oculomotor changes are implemented by various models? The nervous system adapts to changes at multiple timescales (Smith et al., 2006; Kording et al., 2007; Ethier et al., 2008). Instead of using the parameter values from the original paper, we fit variants of this model to the autocorrelation function (see “Methods” for details). These versions only differ in the number of free parameters (i.e., parameters that are not fixed, but found by minimizing the error between the model autocorrelation and the experimental autocorrelation). Our first model assumes all timescales are equally important; this produced the worst fit (*c* = RMS error: 1.3e-4) but used only 1 free parameter for scaling. We also used a model that assumed a distribution of weights across timescales (RMS error: 8.40e-5) for a fit that only had 2 free parameters, one to define a power-law of importance across timescales and one for scaling. All these models can, given the right parameters, provide relatively good fits to the single-day autocorrelation function. Importantly, these multiple timescale models fit the data well, emphasizing the existence of multiple timescales in the distribution of ongoing changes.

**Figure 3:**
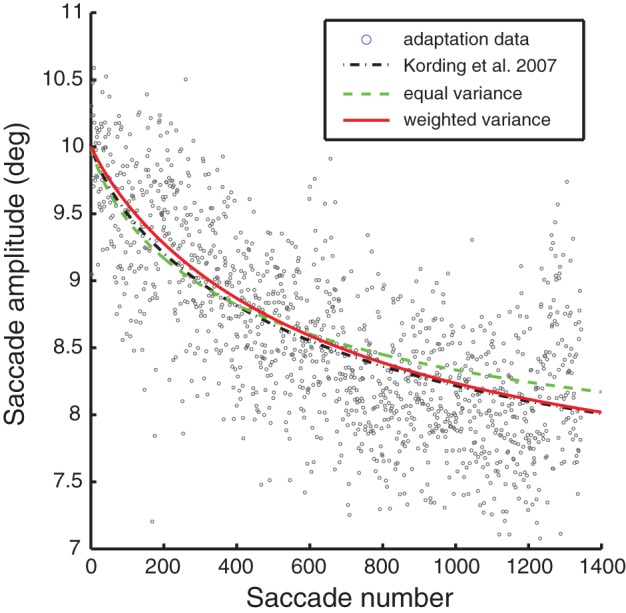
**A representative saccade adaptation session (gray dots) with models represented as in Figure 2.** The only parameter adjusted to fit this data was the observation noise.

Models of optimal adaptation depend not only on the timescale of changes in the motor system, but also on the reliability of the feedback. If the motor system output has a great deal of random variability, this timescale of variation should not be adapted to. As we have no way of directly measuring this observation noise we fit this parameter to the adaptation data from a single session (see Figure 3). Thus, our final model predictions ultimately rely on one-day saccade adaptation data as well as fitting to the lesioned autocorrelation data. Note that all the models lead to good fits for the one-day adaptation data; fits from a single session are not sufficient to visibly distinguish between these different models of adaptation. More formally, BIC would select the equal variance model with 1 fitted parameter over the weighted variance model with 2 fitted parameters (BIC: 2675 < 2695).

**Figure 4:**
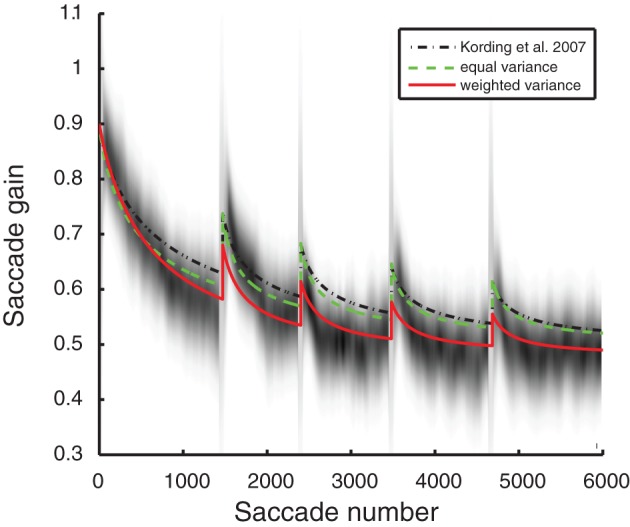
**Model saccade adaptation over multiple days from Robinson et al. (2006).** Gray shading: saccade data from the original experiment. Black line, dotted: original Kording et al. (2007) model. Green, dashed: equal variance model (*a* = 1, see “Methods”) fit to lesioned autocorrelation and unlesioned single-session saccades. Red, solid: weighted variance model fit to lesioned autocorrelation and unlesioned single-session saccades.

Robinson and colleagues obtained recordings in a saccadic gain adaptation experiment that span multiple days and thus many timescales (Robinson et al., 2006). Each of the models of optimal adaptation we have introduced makes a unique prediction of the way adaptation should unfold over multiple days. The full, weighted-variance model produces the best fit of the data while other models fail to simultaneously describe the single-day and multiple-day adaptation effects. The errors in these models were measured by the log likelihood of the data given the means and variances of the experimental saccade data—that is, a function of the probability of the given plot occurring randomly given the means and variances of the experimental saccade adaptation data. Larger values are more likely. The fit of the models, as measured by the log likelihood, for the original KTS model (−33.0 ± 1.2) and the equal variance model (−22.0 ± 0.8) were lower than for the weighted variance model (−6.8 ± 0.2). The weighted variance model also matches the multiple-timescale saccade data better qualitatively, as observed in Figure 4.

It is possible that the improved fit may be a product of introducing one additional parameter for the weighted variance model. To consider this, we used BIC (Schwarz, 1978) to compare the models while correcting for this difference. Even when correcting for the larger number of free parameters, BIC model comparison favors the weighted variance model (BIC_weighted variance_ = 40.5 ± 0.5 compared to BIC_original KTS_ = 84.0 ± 2.4 and BIC_equal variance_ = 62.0 ± 1.7). It appears that a model where adaptation is well matched to measured ongoing changes of the oculomotor system (autocorrelation in Figure 1) best predicts the experimentally measured multi-timescale aspects of saccadic gain adaptation.

## Discussion

Here we have shown that a simple model can capture the spectrum of fluctuations of the oculomotor system of monkeys with lesions in the posterior cerebellar vermis that impair saccadic gain adaptation. When this model is then used to predict optimal adaptation over longer timescales we find that it faithfully replicates the experimental data better than previous models that were not based on knowledge of the fluctuations of the oculomotor system.

The approach that we have taken here needed to make several assumptions that are at best approximations. Our analysis assumes that there is nearly negligible adaptation over the duration of the experiment in monkeys with lesions in the cerebellar vermis which is supported by previous research (Optican and Robinson, 1980). However, there appears to be weak learning at longer timescales (Barash et al., 1999). This could bias the autocorrelation function toward high frequencies as low frequencies are still adapted away. Moreover, our analysis assumed that each timescale contributes independently to changes in the oculomotor system. If the changes were not independent then more complicated, non-linear learning mechanisms would be superior. It would require significantly more data to thoroughly analyze non-linear aspects of fluctuations of the gain of saccades but such characterizations would lead to additional experimentally testable predictions about saccadic gain adaptation.

We show that saccade adaptation rates are well matched to the timescales of fluctuations of the motor plant, but what are the sources of these fluctuations? Fatigue is often cited as a change that requires adaptive compensation, but recent studies show fatigue is likely present but not as critical in the oculomotor system (Prsa et al., 2010) except perhaps in specific, repetitive paradigms (Golla et al., 2008). The primary sources of these fluctuations are likely to be non-motor, internal sources including drowsiness, attentional modulation, neuronal fatigue, etc. (Prsa and Thier, 2011). Importantly, to enable optimal behavior, the nervous system does not need to explicitly identify the source of the disturbances, but rather only respond appropriately to the timescales of those disturbances.

Our research suggests that the timescales of adaptation in the nervous system are well matched to the normal timescales of change in the oculomotor system. Given that saccadic gain adaptation seems to be driven by the cerebellum (Optican and Robinson, 1980) it indicates that the cerebellum has been shaped by the statistics of ongoing changes in the motor plant. There are two ways how this shaping may have occurred. Either over evolutionary timescales the nervous system has acquired an innate knowledge of these timescales or they are learned over our lifetime. Recent research on reaching indicates that such meta-learning is possible (Smith et al., 2006; Burge et al., 2008; Wei and Koerding, 2009). Further research would be necessary to explore the relationship between the evolutionary and learned components of the adaptive response.

We have shown that oculomotor adaptation is well matched to the spectrum of timescales that affect normal, natural changes in the oculomotor plant as well as artificial experimental perturbations. The models that fit well across conditions were better able to predict adaptation over multiple days. Our approach is thus similar in spirit to previous normative approaches to perception (Knill and Richards, 1996; Kording, 2007; Vilares and Kording, 2011). These approaches have shown that salient aspects of sensory systems can be understood assuming that the nervous system is optimally matched to the statistics of natural scenes (Olshausen and Field, 1996; Bell and Sejnowski, 1997) and sounds (Lewicki, 2002). Our research adds to the growing literature showing that through evolution or learning, the nervous system appears to generally be well matched to the properties of our body and the world surrounding us.

### Conflict of interest statement

The authors declare that the research was conducted in the absence of any commercial or financial relationships that could be construed as a potential conflict of interest.
